# Regulatory modules controlling early shade avoidance response in maize seedlings

**DOI:** 10.1186/s12864-016-2593-6

**Published:** 2016-03-31

**Authors:** Hai Wang, Guangxia Wu, Binbin Zhao, Baobao Wang, Zhihong Lang, Chunyi Zhang, Haiyang Wang

**Affiliations:** Biotechnology Research Institute, Chinese Academy of Agricultural Sciences, 12 Zhongguancun South Street, Haidian District Beijing, 100081 China

**Keywords:** Maize, Shade, Phytochrome, Transcriptome, Regulatory module

## Abstract

**Background:**

Optimization of shade avoidance response (SAR) is crucial for enhancing crop yield in high-density planting conditions in modern agriculture, but a comprehensive study of the regulatory network of SAR is still lacking in monocot crops.

**Results:**

In this study, the genome-wide early responses in maize seedlings to the simulated shade (low red/far-red ratio) and also to far-red light treatment were transcriptionally profiled. The two processes were predominantly mediated by phytochrome B and phytochrome A, respectively. Clustering of differentially transcribed genes (DTGs) along with functional enrichment analysis identified important biological processes regulated in response to both treatments. Co-expression network analysis identified two transcription factor modules as potentially pivotal regulators of SAR and de-etiolation, respectively. A comprehensive cross-species comparison of orthologous DTG pairs between maize and *Arabidopsis* in SAR was also conducted, with emphasis on regulatory circuits controlling accelerated flowering and elongated growth, two physiological hallmarks of SAR. Moreover, it was found that the genome-wide distribution of DTGs in SAR and de-etiolation both biased toward the maize1 subgenome, and this was associated with differential retention of various *cis*-elements between the two subgenomes.

**Conclusions:**

The results provide the first transcriptional picture for the early dynamics of maize phytochrome signaling. Candidate genes with regulatory functions involved in maize shade avoidance response have been identified, offering a starting point for further functional genomics investigation of maize adaptation to heavily shaded field conditions.

**Electronic supplementary material:**

The online version of this article (doi:10.1186/s12864-016-2593-6) contains supplementary material, which is available to authorized users.

## Background

A major challenge in modern agriculture is to produce enough food with limited arable land to meet the demand from an ever-growing world population [1]. One effective practice is to increase the crop planting density in order to enhance the yield on a per area unit basis, which has been successfully employed in maize production [2]. A key determinant of the optimum planting density of field-grown maize is its response to shade. At dense stands, maize perceives the shade mainly as a decrease in the red (R):far-red (FR) light ratio caused by the depletion of photosynthetically active radiation (PAR) by neighboring plants. Such a signal serves as a warning of forthcoming competition from neighbors and triggers a number of physiological alterations of maize plants for them to outgrow their competitors, including reallocation of resources into stem elongation from other organs; longer leaves; lowered mechanical strength of stems; elevated ear height; higher lodging rate; early flowering and senescence; reduced photosynthesis efficiency and grain filling; and also active suppression of defense responses against pathogens and herbivores [1, 3–9]. The above-mentioned phenomena, collectively known as the shade avoidance response (SAR), is detrimental to the grain yield of maize as plant density increases.

The molecular components and genetic network controlling SAR have been extensively studied in the dicot model species *Arabidopsis* [10]. The *Arabidopsis* genome encodes five phytochromes (phyA to E) serving as photoreceptors for R and FR light. Among them phyB plays a major role in shade detection [11]. Phytochromes exist in two photo-convertible isoforms: R light triggers the photoconversion of phytochromes from their inactive Pr form to the active Pfr form, while FR light photoconverts the Pfr form back to the Pr form. The Pfr form of phytochormes translocates into the nucleus to trigger genome-wide transcriptional changes and subsequent photo-responses. Shade reduces the Pfr:Pr ratio, and also the nuclear accumulation, of phyB, leading to the accumulation of the E3 ligase CONSTITUTIVE PHOTOMORPHOGENIC1 (COP1) in the nucleus [12] and enhanced 26S proteasome-mediated degradation of several transcription factors, including ELONGATED HYPOCOTYL5 (HY5), HY5-HOMOLOG (HYH), LONG HYPOCOTYL IN FAR-RED1 (HFR1), and LONG AFTER FAR-RED LIGHT1 (LAF1) [13–16]. Recent progress showed that phytochromes could function as transcriptional regulators by interacting directly with numerous transcription factors on the promoters of target genes, conferring rapid responses to light signals [17, 18]. More importantly, phytochromes suppress the shade response by antagonizing a group of bHLH transcription factors termed PHYTOCHROME-INTERACTING FACTORS (PIFs). Shade induces the dephosphorylation and activation of PIF7 and also promotes the protein stability of other PIFs (PIF1/3/4/5) as the result of deactivation of phyB [19–24]. PIFs regulate nearly all physiological aspects of SAR, such as flowering and auxin-dependent elongated growth [23, 25–27]. Shade also swiftly induces the transcript abundance of a number of transcription factors in *Arabidopsis*, including a group of homeobox genes (*HAT1-4* and *AtHB4*) and bHLH family members (*PAR1*, *PAR2*, *HFR1*, *PIL1*, and *PIF6*/*PIL2*), which act as either positive (such as *AtHB2*, *PIL1*) or negative (such as *HFR1*, *PAR1*, and *PAR2*) regulators to fine-tune the progress of SAR [28–32].

Although the genetic and regulatory networks controlling SAR has been extensively studied in *Arabidopsis*, these are far less well defined in monocots, including maize [1]. Maize as well as all other monocots only has three types of phytochromes (phyA to C) [33]. Similar to *Arabidopsis*, the maize SAR is largely mediated by phyB, and the two copies of phyB (phyB1 and phyB2) derived from an ancient tetraploidization event contributes differently to distinct physiological aspects of SAR, indicating both redundancy and sub-functionalization of phyB activities [34]. Dramatic physiological and morphological variations of shade response have been observed in natural populations of maize, and modern maize varieties are generally associated with enhanced shade tolerance under high-density planting conditions [2, 35, 36], thus attenuated SAR might have been historically selected by maize breeders for adaptation to high density planting [1]. A number of domestication genes controlling important agronomic traits have been cloned in maize, such as *teosinte branched1* (*tb1*, controls tillering) and *teosinte glume architecture1* (*tga1*, controls glume development) [37, 38], but none has been identified to regulate SAR hitherto. It is highly likely that the improved shade response in modern maize is a relatively complex trait collectively controlled by multiple loci, considering the complexity of the regulatory network of SAR in *Arabidopsis*. Despite a lack of comprehensive understanding of the genetic architecture of SAR in maize and many other crop species, there has been significant interest in modifying photoperception for crop improvement. For example, overexpression or targeted modification of phyA or phyB has been used to improve plant architecture and increase yield in densely planted crops [39–45]. Other master regulators of shade response downstream of phytochromes such as PIFs, however, have not been utilized as targets in crop SAR engineering.

To improve maize SAR more efficiently, either by molecular engineering or traditional breeding methods, it is necessary to achieve a mechanistic understanding of the molecular components and regulatory networks controlling maize SAR [1]. Here, the maize SAR controlled by phyB signaling, and also the FRc-mediated de-etiolation controlled by phyA signaling, was examined by time-series genome-wide expression profilling. The shade avoidance transcript profile significantly overlapped with that in de-etiolation, with contrasting directions of expression regulation for most overlapping differentially regulated genes in the two experiments. Enrichment analysis of *cis*-elements on light-responsive gene promoters identified binding sites for TFs involved in various signaling pathways that cross-talk with phytochrome signaling. Co-expression network analysis identified two transcription factor modules each functioning in SAR and de-etiolation, and may serve as regulatory hubs in each process. A cross-species comparison of the shade transcriptome between maize and *Arabidopsis* identified both conserved and divergent patterns of expression regulation on various biological processes such as flowering and elongated growth. Moreover, it was found that the two subgenomes of maize differentially retained shade/de-etiolation-responsive genes and *cis*-elements, indicating fractionation of the two subgenomes in light-signaling. This study will be helpful for future functional genomics investigation of maize adaptation to heavily shaded field conditions.

## Results and discussion

### The simulated shade environment

**Fig. 1:**
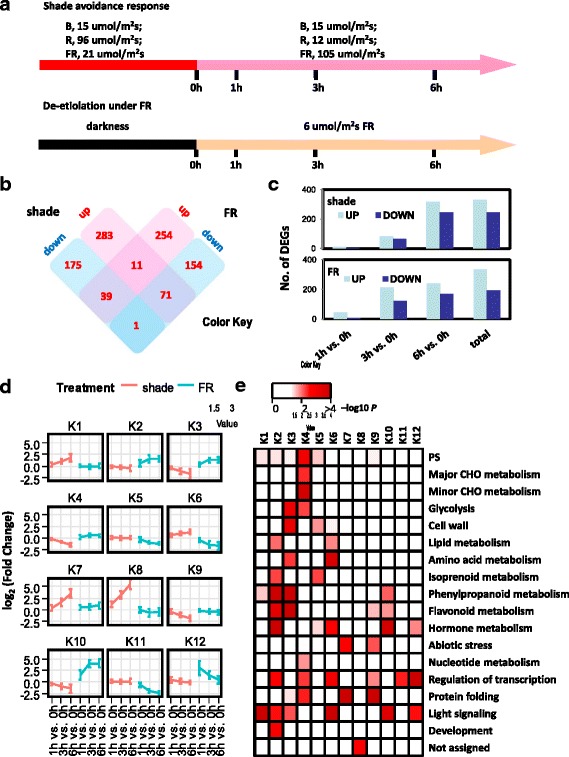
Dynamic progression of maize transcriptome in SAR and FRc-mediated de-etiolation. **a** A schematic representation of the light treatments used in this study. **b** Shared and unique DTGs in SAR and de-etiolation. Note that most overlapping DTGs were regulated in opposite directions in the two experiments. **c** The number of DTGs at various time points in SAR and de-etiolation. **d** The expression profiles of 12 gene clusters identified from 988 DTGs by K-means clustering. **e** Functional category enrichment (modified MapMan bins) among the 12 clusters

Genome-wide transcriptional dynamics of the shade avoidance response (SAR) has been extensively studied in the model dicot *Arabidopsis* [46–48], but less well defined in economically important monocots. This study aimed to dissect the regulatory network controlling the maize SAR, a process believed to be attenuated by intense artificial selection during domestication and breeding to avoid the adverse effects of SAR under densely planted field conditions [1]. A simulated shade condition identical to that used before in *Arabidopsis* [46] was adopted to facilitate direct comparison between the two species (Fig. 1a). Maize seedlings grown under the simulated shade condition displayed phenotypes characteristic of SAR, including elongated leaves and less anthocyanin accumulation, compared to those grown under high R/FR (Additional file 1: Figure S1), indicating the validity of such an experimental condition.

### Dynamic transcriptional reprogramming in the maize shade avoidance response

To analyze the dynamics of the SAR at a genome-wide scale, global gene expression profiles upon 1 h, 3 h and 6 h exposure to low R/FR were inspected. A transcriptional profiling on maize de-etiolation under FRc was also conducted (Fig. 1a), a process sharing common regulatory components with SAR [47]. Principal component analysis (PCA) showed high degree of reproducibility among biological replicates within each group (Additional file 2: Figure S2). A total of 580 genes were identified as differentially regulated in response to the simulated shade within 6 h (absolute fold change > =2 and FDR adjusted *p*-value < =0.05), with 333 up-regulated genes and 247 down-regulated genes. Comparable numbers of DTGs were found in FR-mediated de-etiolation, with 336 genes up-regulated and 194 down-regulated (Fig. 1b). Due to the partial overlap between the two DTG sets, in total 988 early-responsive DTGs in response to shade and/or FR were observed, representing 2.5 % of the maize transcriptome (Summarized in Additional file 3: Table S1). Validation of the expression levels of 20 genes by qRT-PCR showed a high correlation (R^2^ = 0.938) between RNA-seq and qRT-PCR (Additional file 4: Table S2).

The number of DTGs increased with different dynamics in the two experiments. Most DTGs occurred between 3 h and 6 h in SAR, but between 1 h and 3 h in de-etiolation, suggesting faster responses of maize transcriptome in de-etiolation (Fig. 1c). There was only a limited overlap between shade- and FR-responsive gene sets (122 out of the 988 DTGs were present in both DTG sets), and most common DTGs (110 out of the 122 common DTGs) were regulated in opposite directions in SAR and de-etiolation (Fig. 1b). In agreement with this, there was a negative correlation in the expression pattern of these 110 common DTGs between SAR and de-etiolation, with Pearson correlation coefficients of −0.57, −0.53 and −0.34 for 1 h vs. 0 h, 3 h vs.0 h, and 6 h vs. 0 h, respectively. Genes with such expression patterns were also reported in *Arabidopsis* [47]. Such phenomenon is expected since according to the model established in *Arabidopsis*, SAR and FR-mediated de-etiolation are initiated mainly by inactivation of phyB and activation of phyA, respectively, and these two classes of phytochromes share overlapping downstream signaling cascades [49]. Therefore, DTGs regulated in opposite directions are likely primary targets downstream of phytochromes. In support of this notion, the aforementioned negative correlation weakened with time, indicating a gradual increase of secondary effects downstream of phytochrome signaling cascades.

**Fig. 2:**
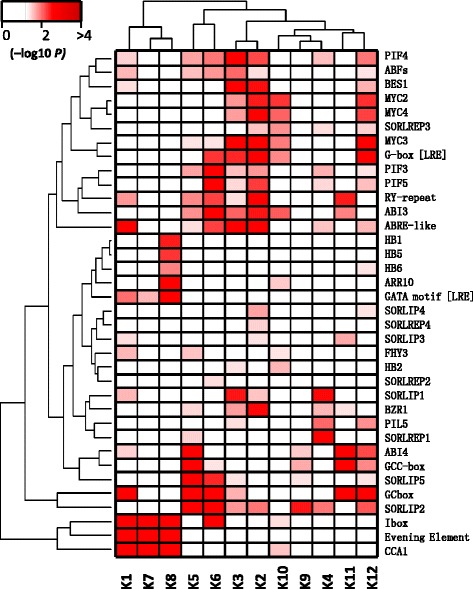
Enriched *cis*-elements in each DTG cluster. For each DTG cluster, the genomic regions from 2 kb upstream to 0.5 kb downstream of the transcription start site were assessed for their over-representation of plant *cis*-elements

To gain an insight into the biological processes involved in maize SAR and de-etiolation, the 988 DTGs were grouped using the K-Means clustering algorithm embedded in MapMan [50], and 12 gene clusters (K1-K12) were identified (Fig. 1d and Additional file 3: Table S1). All clusters showed enrichment for one or more MapMan bins (functional categories) (Fig. 2d). For example, genes involved in photosynthesis, major and minor CHO metabolism, glycolysis, transcriptional regulation and protein folding were enriched in K4, which contained shade-repressed genes. Notably, the K8 cluster, which represents genes that were highly induced by shade and therefore of particular interest, were enriched for genes unassigned with any functions. It was caused by the fact that all the 47 genes in K8 do not have functionally characterized orthologs in other organisms except two orthologs of *Arabidopsis FLAVIN-BINDING, KELCH REPEAT, F-BOX1* (*FKF1*) (Additional file 3: Table S1). During FR-mediated de-etiolation, early-induced genes (K12, induced within 1 h FR treatment) and late-induced genes (K10) showed differential enrichment of MapMan bins. Although they were both enriched for genes involved in light signaling and hormone metabolism, K12 was particularly enriched with transcription factors while K10 was enriched for genes of phenylpropanoid and flavonoid metabolism pathways, suggesting the importance of transcription factors as early primary targets of phyA signaling in maize.

### Differentially enriched cis-elements in distinctly light-regulated gene clusters

Genes with similar expression patterns are likely to share common regulatory *cis*-elements in their promoters, thus the enrichment of *cis*-elements for each DTG cluster was analyzed. This led to the identification of a total of 119 *cis*-elements enriched in at least one DTG cluster (Additional file 5: Table S3). *Cis*-elements enriched in more than 6 clusters or with functions associated with light signaling were shown in Fig. 2. K7, K8 and K1 represent shade-induced DTGs (Fig. 1d). One striking common feature of them is significant enrichment of I-box, the evening element, CCA1 binding site, and the light-responsive GATA motif. In addition, the K8 genes, which were most highly induced by shade, were enriched with the binding sites for a group of HB transcription factors, including HB1/5/6 and ARR10, consistent with the finding in *Arabidopsis* that HB family members were among the early shade-induced genes [3]. Notably, several enriched motifs for K1, K7 and K8 genes, such as the light-responsive GATA motif, ARR10 binding site and GCbox, were not enriched on the promoters of shade-responsive *Arabidopsis* genes [46], suggestive of the unique functions of corresponding transcription factors in maize SAR. K12 and K10 genes, which represent early and late FRc-induced DTGs, respectively, were enriched with G-box motifs, especially binding sites for MYC2/3/4, implying a potential role of JA pathway components in FR-mediated de-etiolation. In addition to the binding sites of MYC2/3/4, *cis*-elements related to various other hormone pathways were found enriched in the DTG set, such as those related to auxin (ARF binding site motif), GA (ARR10 binding site motif) and BR (binding sites of BES1 and BZR1) (Additional file 5: Table S3 and Fig. 2), suggesting combinatorial complexity of these *cis*-elements together with light-regulated *cis*-elements on the transcriptional regulation of target genes. To investigate whether functionally related *cis*-elements might have similar enrichment patterns, *cis*-elements were hierarchically clustered based on their pattern of enrichment across all DTG clusters (Fig. 2). Transcription factor binding motifs with similar sequences tend to group together, such as PIL5/BZR1 and PIFs/MYCs. It was reported that over half of PIL5 target genes are also targets of BZR1 [51], but the integration of other transcription factors on light controlled promoters still awaits further investigation. Despite the abundance of G-box like elements in this analysis, enrichment of HY5 binding sites was not observed in any DTG cluster, consistent with the fact that *HY5* transcript abundance was not significantly altered during SAR in this study. In *Arabidopsis*, *HY5* was initially considered to play no roles in SAR under simulated shade conditions [47]. But it was later found that *HY5* was late induced by shade, and HY5 binding sites were weakly enriched in shade-responsive genes after prolonged (>1 d) shade treatment [46]. And two recent studies identified *HY5* as a repressor of SAR when a sunfleck interrupts shade light on a daily basis [52] and controls hypocotyl and leaf morphology in response to shade [53]. A lack of HY5 binding site enrichment in this dataset suggests a limited role of *HY5* in the early signaling events of SAR in maize, or distinct recognition sites of maize HY5 compared to *Arabidopsis* HY5.

### Changing inventories of transcription factors in SAR and de-etiolation

**Fig. 3:**
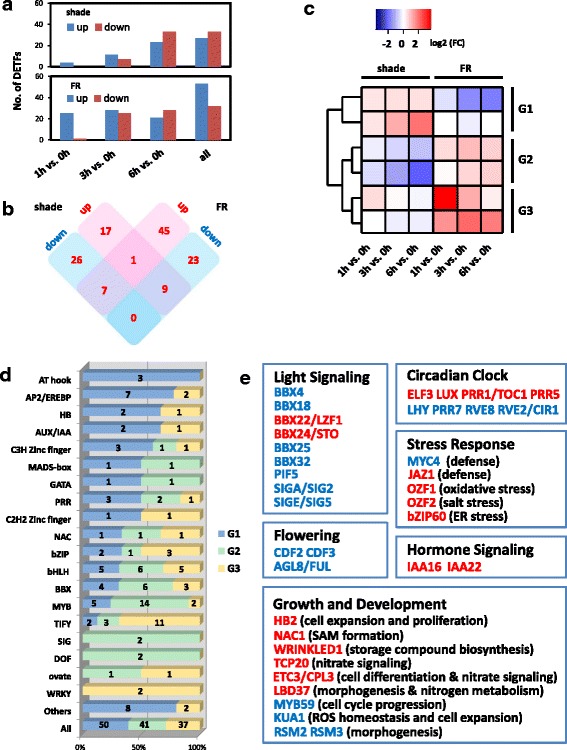
The dynamics of light-regulated DTTFs. **a** The number of DTTFs at various time points in SAR and de-etiolation. **b** The venn diagram showing shared and unique DTTFs in SAR and de-etiolation. **c** The 128 DTTFs were clustered into three groups (G1,G2 and G3) using the Self Organization Tree Algorithm (SOTA). **d** Distribution of TF familys among the three groups of TFs. The number of genes in each family is shown. **e** Representative functions and TFs differentially regulated in response to shade. TFs shown in red were shade-induced while those in blue were shade-suppressed. For clarity, maize TFs were shown as their *Arabidopsis* orthologs

Most functionally characterized domesticated genes are transcription factors [54, 55], and it was suggested that maize SAR has been targets of domestication in order for maize to adapt to the high-density stands in modern field conditions [1, 40]. Thus special attention was paid to TFs that are transcriptionally regulated, and are therefore potentially involved, in SAR. To identify TFs in our DTG sets, DTGs with homology to known TFs were combined with information from the GRASSIUS database, followed by manual removal of mis-annotated genes in GRASSIUS (Additional file 6: Table S4). Among the 580 shade-responsive DTGs, 27 up-regulated and 33 down-regulated differentially transcribed TFs (DTTFs) were identified. A larger proportion of DTGs in de-etiolation was identified as transcription factors (53 up-regulated and 32 down-regulated DTTFs among 530 DTGs) (Fig. 3a), but this set of DTTFs only shared limited overlap with that of SAR. More DTTFs were regulated in opposite directions than those regulated in the same direction in SAR and de-etiolation, a scenario also observed for DTGs (Fig. 3b). Interestingly, in contrast to the previous report that over 30 *Arabidopsis* TFs displayed over two fold induction or repression within 1 h of shade treatment, only 4 up-regulated TFs were found in maize at 1 h of the same treatment, including *ZmHB53* (orthologous to *Arabidopsis HB2/HAT4*, a marker gene for SAR [56, 57]), *ZmLBD6* (orthologous to *Arabidopsis LBD37,* which controls leaf morphogenesis and nitrogen metabolism [58]), *ZmMYBR97* (a putative ortholog of *Arabidopsis ENHANCER OF TRY AND CPC 3* (*ETC3*), a regulator of epidermal cell differentiation and nitrate signaling [59, 60]), and *ZmGLK54* (a putative ortholog of *Arabidopsis LUX*). Nevertheless, maize TFs responded to FR rather swiftly: 25 up-regulated and one down-regulated transcription factors were identified from the 52 early-responsive DTGs at 1 h FR treatment (Fig. 3a). This is comparable to the scenario in *Arabidopsis*, in which over 30 % of the early-responsive genes in FR-mediated photomorphogenesis were transcription factors, representing a master set of genes that orchestrate the expression of the downstream targets in the phyA-directed transcriptional network [61].

**Fig. 4:**
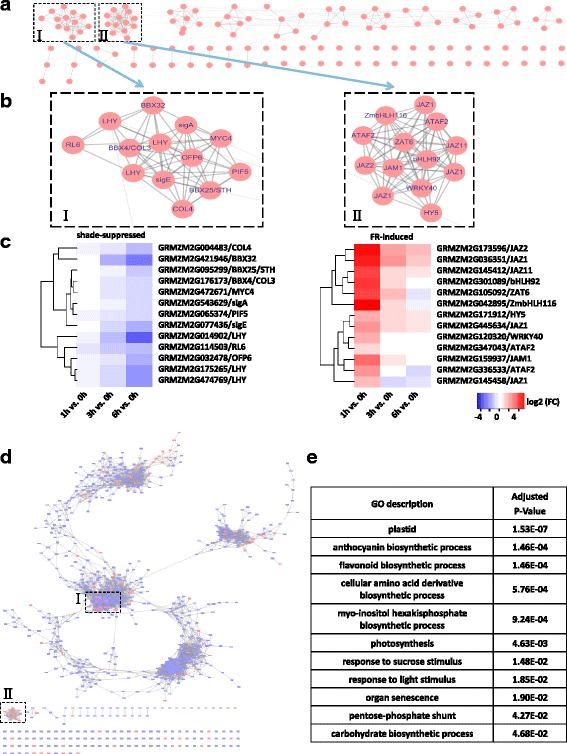
Transcription factor modules in SAR and FRc-mediated de-etiolation. **a** Two densely connected transcription factor groups (Module I and II) each containing 13 TFs were identified in the co-expression TF network using the Markov cluster algorithm (MCL).**b** A close-up view of the two modules. Genes with functionally characterized *Arabidopsis* orthologs were labeled with the names of their *Arabidopsis* counterparts for clarity except ZmbHLH116. **c** Gene expression heatmaps of Module I in SAR and Module II in de-etiolation. **d** Module I and II in the context of a co-expression network containing all the 988 DTGs. TFs were shown in orange. **e** Enriched GO terms of first neighbors of Module I

To detect potential family-specific expression trends, DTTFs were clustered using the Self Organization Tree Algorithm (SOTA) based on their expression pattern in SAR and de-etiolation, and further categorized into 3 groups (G1-G3) (Fig. 3c). G1 and G2 represent TFs that were induced or repressed by shade, respectively. Some TF family members preferably or exclusively belong to G1 and/or G2, such as AT hook containing TFs, AP2/EREBP, MADS, GATA, PRR, MYB, SIG and DOF. TFs that were non-responsive to shade, but nevertheless FR-inducible, comprised G3. TIFY and WRKY TFs preferentially belong to G3, and are therefore more functionally related to phyA signaling during de-etiolation (Fig. 4d).

Homology-based function prediction of DTTFs in maize SAR showed many biological processes potentially regulated by these DTTFs, including light signaling, circadian clock, flowering, stress response, hormone signaling, as well as growth and development (Fig. 3e). Eleven shade-regulated *BBX* family members were identified, including 3 shade-induced (orthologous to *Arabidopsis BBX12*, *BBX22*, and *BBX24*) and 8 shade-repressed genes (orthologous to *Arabidopsis BBX4, BBX5*, *BBX18*, *BBX25*, and *BBX32*). In *Arabidopsis*, *BBX22* negatively regulates SAR [62, 63], while *BBX24* promotes SAR by impairing DELLA activity [64]. Moreover, *BBX18* and *BBX25* promote hypocotyl elongation under a low R/FR ratio [65, 66]. The roles of other *BBX* genes in SAR have not been defined, although *BBX4* and *BBX32* were identified as positive and negative regulators of photomorphogenesis, respectively [67, 68].

Plastid was considered as an important player in light signaling [69], and two putative chloroplast localized sigma factors (SIGA/SIG2 and SIGE/SIG5) were found shade-repressed. SIGA, but not SIGE, was also reported as a shade-repressed gene in *Arabidopsis* [46]. Moreover, *Arabidopsis* SIGA and SIGE expression displayed phytochrome-dependent induction by R or FR illumination during de-etiolation, and function as positive and negative regulators of photomorphogenesis, respectively [70–72].

TFs with functions in circadian clock and flowering were also among shade regulated DTTFs. Orthologs of *ELF3*, *LUX*, *TOC1*, and *PRR5* were up-regulated while those of *LHY*, *PRR7*, *RVE2*, *RVE8* were down-regulated. Two Dof-type zinc finger genes orthologous to *Arabidopsis CDF2* and *CDF3* were down-regulated by shade. *Arabidopsis* CDF2 and CDF3 repress *CO e*xpression to delay flowering and such repression is antagonized by the GI/FKF1 complex [73, 74]. Interestingly, in addition to the shade-repressed *CDF2/3* expression, strong induction of the two *GI* and two *FKF1* orthologs in maize in response to shade was observed. Therefore, the mechanism of shade-induced early flowering might be conserved between maize and *Arabidopsis*.

Shade also influenced many aspects of growth and development in maize. Three DTTFs potentially involved in nitrogen signaling were identified, including orthologs of *Arabidopsis TCP20* [75], *ETC3* [59], and *LBD37* [58]. Notably, *ETC3* and *LBD37* are two of the four extremely early shade-responsive DTTFs (induced within 1 h), suggesting the importance of nitrogen signaling in early maize SAR. Other shade-induced up-regulated genes include an ortholog of *Arabidopsis WRINKLED1*, which controls biosynthesis of triacylglycerol, an important storage compound [76]; an ortholog of *AtNAC1*, which controls SAM formation [77]; and an *HB2* ortholog potentially involved in cell expansion and proliferation. Moreover, shade suppressed the expression of *ZmMYB88*, an ortholog of *ATMYB59*, which functions to regulate cell cycle progression [78]; *ZmMYBR35*, an ortholog of *KUA1*, which is essential for cell expansion and ROS homeostasis [79]; and four single-MYB transcription factor genes orthologous to *Arabidopsis RADIALIS-LIKE SANT/MYB 2* (*RSM2*) and *RSM3*. Although *RSM2* and *RSM3* have not been functionally studied, *RSM1* overexpression plants are hypersensitive to red light during de-etiolation, characterized by a shorter hypocotyl than wild type plants [80].

A number of TFs related to stress response were also identified, including the upregulation of two *Oxidation-related Zinc Finger* (*OZF*) genes orthologous to *Arabidopsis OZF1* and *OZF2*, positive regulators of the oxidative stress response and salt stress response, respectively [81, 82]; and also *ZmbZIP112*, an ortholog of *Arabidopsis bZIP60*, which regulates unfolded protein response (UPR) in ER stress [83]. Moreover, two JA signaling components were differentially regulated, including up-regulation of a *JAZ1* ortholog and down-regulation of a *MYC4* ortholog.

In *Arabidopsis*, a large number of shade-induced TFs have been identified and functionally characterized, including a group of homeobox genes (*HAT1-4* and *ATHB4*) and bHLH family members (*PAR1*, *PAR2*, *HFR1*, *PIL1*, and *PIF6/PIL2*), which function as either positive (such as *ATHB2*, *PIL1*) or negative (such as *HFR1*, *PAR1*, and *PAR2*) regulators of SAR [28–31]. Phylogenetic analysis of homeobox TFs in *Arabidopsis* and maize identified *ZmHB53* and *ZmHB78* as two orthologs of the five shade-induced *Arabidopsis HB* genes, indicating an expansion of this small *HB* family clade in *Arabidopsis* after its divergence from maize (Additional file 7: Figure S3). *ZmHB53* was an extremely early shade-induced gene up-regulated by shade within 1 h, and it was found down-regulated in de-etiolation. *ZmHB78* was unresponsive to any light treatment in this study. *Arabidopsis PAR1* and *PAR2* are two atypical bHLH TFs, and only one maize ortholog was found (GRMZM2G364528) (Additional file 8: Figure S4). However, this gene was not up-regulated by shade, although its down-regulation in de-etiolation was observed. *PIF*s and *PIL*s, together with an atypical bHLH gene *HFR1*, comprise a small bHLH clade that are crucial components of SAR. Phylogenetic analysis showed that seven maize bHLH proteins were orthologous to *Arabidopsis* PIF1/3/4/5, all characterized by an N-terminal APB motif responsible for their interaction with phyB (Additional file 9: Figure S5). Although regulation of PIF activity mainly occurs at the protein level in *Arabidopsis*, two light-regulated maize *PIFs*, *ZmbHLH47* (shade-suppressed) and *ZmbHLH60* (FR-induced), were identified. For *HFR1*, *PIL1* and *PIL2*, however, no obvious orthologs were detected in maize, suggesting their fast evolution and expansion in *Arabidopsis* or a loss of these TFs in maize.

The dynamics of DTTFs during FRc-mediated de-etiolation also suggests complex cascades of transcriptional control. Interestingly, up-regulation of a number of JA pathway signaling components, including 15 *JAZs*, 1 *MYC2* ortholog, and 1 *JAM3* ortholog, was observed. The integration of phyA signaling and JA pathway has been a field of intense investigation [84]. *MYC2* functions synergistically with *SPA1* to suppress FR-mediated photomorphogenesis [85]. The role of *JAZ* genes in photomorphogenesis is unclear, but JAZ1 protein was reported to be destablized in FRc-mediated de-etiolation and stablized during SAR, and ectopic overexpression of *JAZ1* resulted in exaggerated SAR [7, 86]. In *Arabidopsis,* JA was reported to positively regulate the phenylpropanoid pathway and promote biosynthesis of flavonoids (especially anthocyanin) in a phyA-dependent manner [87], and genes involved in phenylpropanoid and flavonoid metabolism were enriched in K2, K3 and K10, clusters containing FR-induced genes in de-etiolation (Fig. 1e). Consistently, several TFs involved in phenylpropanoid biosynthesis were induced during de-etiolation, including the maize *r1/colored 1* gene orthologous to *Arabidopsis GLABROUS 3* (*GL3*), a maize ortholog of *Arabidopsis IAA26*/*phytochrome-associated protein1* (*PAP1*), three orthologs of *HY5*, and *ZmMYB40*/*ZmMYB-IF35*, an ortholog of *Arabidopsis MYB12*. In *Arabidopsis*, PAP1 is a phytochrome interacting protein and regulates phyA-induced *chalcone synthase* (*CHS*, EC: 2.3.1.74) expression through *HY5. ZmMYB-IF35* has not been functionally characterized, but its ortholog *MYB12* is a transcriptional activator of *CHS*, *flavanone 3-hydroxylase* (*F3H*, EC: 1.14.11.9), *flavonol synthase* (*FLS*, EC: 1.14.11.23) and *chalcone flavanone isomerase* (*CHI*, EC: 5.5.1.6) [88]. In *Arabidopsis*, the promotion of *MYB12 e*xpression is dependent on *HY5* [89], consistent with the role of *HY5* as a positive regulator of anthocyanin biosynthesis [90]. It’s still unknown whether the HY5-MYB12 axis is conserved in maize, despite the up-regulation of both *HY5* and *MYB12* orthologs during de-etiolation in our dataset.

### Co-expression analysis shows possible modularized regulatory hubs in SAR and de-etiolation

To identify co-regulated transcription factor modules that may function as core regulators of maize light signaling, a co-expression correlation network was constructed according to Pearson correlation values between TFs, and densely connected TFs (modules) were identified using the Markov cluster algorithm (MCL) (Fig. 4a). Two modules were identified at a cut-off of 10 for node numbers, and they were found each involved in SAR and de-etiolation (Fig. 4b,c). Module I consists of 13 TFs whose expression levels gradually decreased throughout the shade treatment, including four *BBX* genes, three orthologs of *Arabidopsis LHY*, two sigma factors, as well as orthologs of *Arabidopsis PIF5*, *MYC4*, *RL6,* and *OFP6*; Module II contains 13 TFs that are early-induced (within 1 h) during FRc-mediated de-etiolation, but the inductive effect of FRc on these TFs was later alleviated. Interestingly, most of them have been implicated in stress responses, such as several JA-pathway components (five *JAZ* genes and one *JAM1* ortholog), and also orthologs of *Arabidopsis WRKY40*, *ZAT6, ATAF2*, *bHLH92,* all involved in defense response to biotic and/or abiotic stresses [91–94].

Co-regulated genes may function in concert in specific biological processes, making it possible to divulge the biological functions of the above mentioned TF modules by placing them in the context of a genome-wide co-expression network. The co-expression analysis was extended to all 988 DTGs identified in this study (Fig. 4d). To identify biological processes most closely linked to TF modules, the modules were considered as single nodes in the graph and their first neighbors were subjected to GO enrichment analysis. Ninety-three first neighbors of Module I were enriched with diverse GO terms including “plastid”, “photosynthesis”, terms related to primary and secondary metabolism (such as flavonoid, anthocyanin, cellular amino acid derivatives and myo-inositol hexakisphosphate biosynthesis), as well as terms involved in responses to environment changes (“response to light stimulus” and “response to sucrose stimulus”), suggesting that Module I TFs play regulatory roles in these processes (Fig. 4e). The Module II, however, appeared rather isolated in the co-expression network, and only harbors two first neighbors: GRMZM2G031432 (encoding GA2OX1) and GRMZM2G325683 (encoding an unknown protein). The scarcity of first neighbors was probably due to the fact that Module II TFs responded to FRc much faster than most other genes, leading to relatively low correlation coefficients between these TFs and other genes. The role of *GA2OX* genes in de-etiolation has been reported in *Pisum sativum*, in which HY5 positively regulates the GA catabolism gene *GA2OX2* to reduce the levels of active GAs and suppress the elongated growth in skotomorphogenesis [95]. The maize *GA2OX1* gene is another GA catabolism gene, and the maize *HY5* gene in Module II possibly up-regulated the *GA2OX1* gene expression. Moreover, although HY5 was largely regulated at the protein level, up-regulation of maize *HY5* expression during de-etiolation was observed.

### Cross-species comparison of transcriptional profiles in SAR identifies auxin biosynthesis as a potential target of maize domestication

Although monocots and dicots have split around 140 ~ 200 million years ago, they share a number of common morphological and physiological responses in their shade avoidance syndrome, such as elongated growth and accelerated flowering. Thus we compared shared patterns of expression across all orthologous maize-*Arabidopsis* gene pairs in response to shade. Putative orthologs of shade-responsive maize genes in *Arabidopsis w*ere obtained from the Gramene database, and their expression patterns under shade were retrieved from two previous studies [46, 48]. 174 of the 333 shade-induced maize genes have orthologs in *Arabidopsis*. Among these 174 maize genes, 20 have orthologous *Arabidopsis* genes that are also induced by shade. 182 out of 247 shade-repressed maize genes have their orthologous counterparts in *Arabidopsis*. However, only 11 of these maize genes have *Arabidopsis* orthologs that are shade-repressed (Additional file 10: Table S5). These results indicate that the regulation of shade-induced genes is more conserved than that of shade-repressed genes. Interestingly, 19 orthologous gene pairs displayed contrasting expression patterns in response to shade, suggesting that the biological processes controlled by these genes may be distinctly regulated in maize and *Arabidopsis* during SAR (Additional file 11: Table S6).

**Fig. 5:**
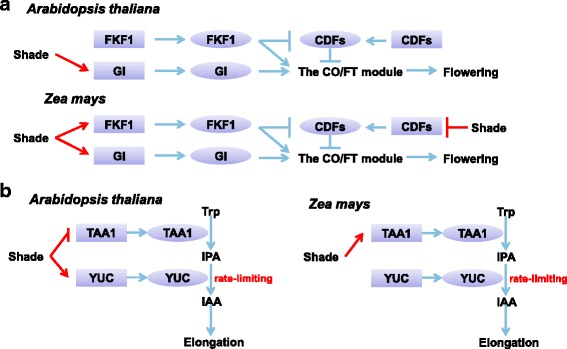
A cross-species comparison of the regulatory circuits controlling flowering and elongation in SAR between *Arabidopsis* and maize. **a** In *Arabidopsis*, the FKF1-GI module positively regulates the CO/FT module activity and flowering by multiple mechanisms. The FKF1/GI complex directly promotes *CO* expression on the *CO* promoter. FKF1 also directly enhances CO protein stability, or suppresses CDF transcription factors (mostly CDF1) to alleviate their repressive effect on *CO* expression. Moreover, GI was reported to directly promote *FT* expression. The *Arabidopsis GI* was reported to be a shade-inducible gene. In maize shade response, however, up-regulation of *FKF1* in addition to *GI* and also the down-regulation of two *CDF*s were observed. **b** A recently revised model of auxin biosynthesis placed YUCCAs downstream of TAA1, with YUCCAs as the rate-limiting enzymes. In *Arabidopsis*, shade leads to strong induction of *YUCCA*s and a slight down-regulation of *TAA1*, resulting in excessive biosynthesis of auxin and elongated growth under shade. In maize, however, only a mild up-regulation of *TAA1* and no obvious alterations in *YUCCA* expression were observed

Special attention was paid to genes controlling elongated growth and accelerated flowering, two hallmarks of SAR. Two flowering promoting genes, *GI* and *FT*, were shade-induced in both species (Additional file 10: Table S5). In *Arabidopsis, GI* and *FKF1* promote the CO/FT module activity by multiple mechanisms (Fig. 5) [74, 96–100]. Although *FKF1* was not a shade-induced gene in *Arabidopsis*, strong shade induction of the two *FKF1* orthologs in maize was observed (Additional file 3: Table S1, Fig. 5a). Shade also repressed the expression of two maize *CDF*s, which are suppressed by FKF1 and negatively regulate *CO* expression in *Arabidopsis*. These results suggested a conserved GI/FKF1-CO/FT axis promoting flowering in SAR, although the regulation of *FKF1* and *CDFs* may have distinct mechanisms in the two species.

Elongated growth is another hallmark of SAR. In *Arabidopsis*, this is achieved by shade-induced rapid biosynthesis of auxin dependent on *TRYPTOPHAN AMINOTRANSFERASE OF ARABIDOPSIS 1* (*TAA1*) [101] and *YUCCA* genes [102]. A recently revised model of auxin biosynthesis pathway placed *YUCCA* genes downstream of *TAA1* [103]. In *Arabidopsis*, shade strongly induces *YUCCAs* but also exerts a weak suppression effect on *TAA1* expression [101]. As YUCCAs catalyze the rate-limiting step, the biosynthesis of auxin is strongly enhanced in response to shade. In maize, although up-regulation of a *TAA1* ortholog was observed, the expression of *YUCCA* genes was not obviously altered (Fig. 5b). Furthermore, in contrast to the previous report that shade-responsive genes in *Arabidopsis* were particularly enriched with the GO term “response to auxin stimulus” [46], this and other auxin-related GO terms were not enriched in shade-responsive maize genes (Additional file 12: Table S7), consistent with the distinct regulatory patterns of auxin biosynthetic genes in the two species. It is possible that the unresponsiveness of *YUCCAs* to shade is due to artificial selection for attenuated SAR in maize, but confirmation of such a hypothesis still awaits further investigation of *YUCCA* expression patterns in the undomesticated ancestors of maize.

### Biased distribution of DTGs toward maize1 is associated with fractionation of cis-elements between the two maize subgenomes

**Fig. 6:**
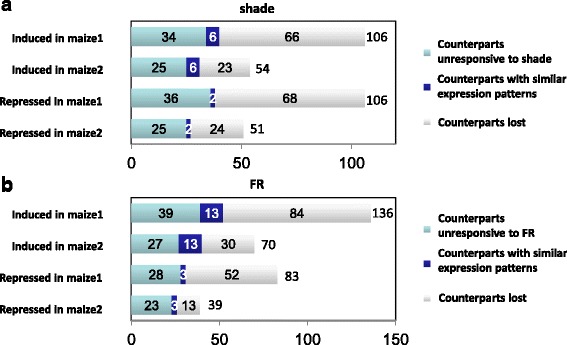
Fraction of the two maize subgenomes in SAR and de-etiolation. **a** Shade-induced and shade-repressed genes unambiguously assigned to the two subgenomes of maize were each categorized into three groups according to the expression patterns and presence/absence of their counters in the other subgenome. **b** The same analysis as in (**a**) on DTGs in FRc-mediated de-etiolation

An ancient tetraploidy event 5–12 million years ago gave rise to the two subgenomes of maize, with the maize1 subgenome experiencing less gene loss and displaying generally higher levels of gene expression compared to maize2 [104], but it’s still unknown whether the two subgenomes respond to environmental stimuli differently. To study the response of the two subgenomes in SAR and de-etiolation, the distribution of DTGs in the two subgenomes was investigated. 212 and 105 DTGs in SAR could be unambiguously assigned to maize1 and maize2, respectively, indicating more dynamic transcriptomic changes in maize1 (*p* < 0.001 in chi-squared test, with the null hypothesis that DTGs fall into maize1 and maize2 with an equal probability) (Fig. 6a, Additional file 13: Table S8). In each subgenome, induced or repressed DTGs were categorized according to their counterparts (homologs) in the other subgenome: 1) counterpart not differentially expressed; 2) counterpart with a similar induction/repression pattern; or 3) counterpart lost after tetraploidy (fractionation). The number of genes falling into the second group should be equal between the two subgenomes. Genes in the third group in maize1 outnumbered those in maize2, which is expected since maize2 suffered from more severe gene loss than maize1. Interestingly, distribution of the first group genes significantly biased toward maize1, for both up- and down-regulated DTGs. Similar gene distribution was also observed for de-etiolation under FRc (Fig. 6b, Additional file 13: Table S8). In sum, the higher responsiveness to shade/de-etiolation for maize1 is due to both higher gene retention rate in maize1, and also higher retention rate of light-responsiveness in existing genes.

The reduced responsiveness of maize2 to light stimuli might be indicative of fractionation of light-regulated *cis*-elements between the two subgenomes. To test this hypothesis, it was investigated whether *cis*-elements enriched in our DTG sets were differentially retained in maize1-maize2 gene pairs. A “shade-induced gene pair” was defined as a pair of homologs each belonging to a subgenome, with at least one of them shade-induced. Enrichment of *cis*-elements was evaluated separately for maize1 and maize2 members of all “shade-induced gene pairs”. As shown in Table 1, distribution of 3 *cis*-elements were found strongly biased toward maize1, including the Evening Element (EE), FUS3 binding site, and SORLREP3. The strongest bias was observed for EE, indicating a significant loss of EE in maize2. In de-etiolation, enrichment of Z-box, AGL3 binding site, ABRE-like binding site, PIF4 binding site, and BZR1 binding site was significantly biased toward maize1. Interestingly, enhanced enrichment of several *cis*-elements in maize2 was also observed, such as the CDC5 binding site in shade-induced gene pairs and the CCA1 binding site in FR-induced gene pairs, suggesting that these *cis*-elements were not involved in gene-induction in this study, or that they function to suppress gene-induction in SAR and de-etiolation, respectively. Differential distribution of *cis*-elements was also found for shade-suppressed and FR-suppressed genes (Table 1). Taken together, these results indicated that fractionation of *cis*-elements contributed to the differential responses of the two subgenomes of maize to light signals.

**Table 1:** Biased retention of *cis*-elements enriched in light-responsive genes between maize1 and maize2

Shade-induced gene pairs					Shade-repressed gene pairs				
ID	maize1		maize2		ID	maize1		maize2	
	*p*.value	motif. prop	*p*.value	motif. prop		*p*.value	motif. prop	*p*.value	motif. prop
Evening Element^a^	2.55E-06	0.200	2.99E-03	0.099	HSEs^a^	1.18E-03	0.133	9.15E-01	0.012
FUS3^a^	9.19E-04	0.152	9.93E-01	0.034	ABFs^a^	4.60E-03	0.117	2.63E-01	0.058
SORLREP3^a^	7.70E-03	0.168	1.00E + 00	0.017	PIL5^b^	3.83E-01	0.042	7.01E-05	0.140
CDC5^b^	3.68E-01	0.032	2.03E-03	0.144	EmBP-1^b^	1.77E-01	0.025	1.92E-05	0.116
FR-induced gene pairs					FR-repressed gene pairs				
ID	maize1		maize2		ID	maize1		maize2	
	*p*.value	motif. prop	*p*.value	motif. prop		*p*.value	motif. prop	*p*.value	motif. prop
Z-box^a^	7.34E-04	0.106	4.17E-01	0.025	CBF2^b^	1.31E-01	0.043	2.79E-03	0.118
AGL3^a^	3.49E-03	0.114	9.29E-01	0.042	GBF1/2/3^b^	1.31E-01	0.043	2.79E-03	0.118
ABRE-like^a^	3.71E-04	0.106	2.42E-02	0.051	DPBF1/2^b^	5.06E-01	0.029	5.64E-03	0.129
PIF4^a^	5.40E-04	0.136	1.21E-02	0.068	PIF5^b^	9.22E-01	0.058	7.23E-03	0.165
BZR1^a^	7.26E-03	0.136	1.23E-01	0.068	PIF3^b^	9.30E-01	0.029	5.25E-03	0.118
CCA1^b^	4.57E-01	0.098	4.85E-03	0.237					

^a^significantly higher enrichment in maize1 than in maize2. ^b^significantly higher enrichment in maize2 than in maize1

## Conclusions

In this study, maize SAR and FRc-mediated de-etiolation was transcriptionally profiled, leading to the identification of important biological processes and *cis*-elements involved in the response to the two treatments. Co-expression network analysis identified transcription factor modules that may serve as pivotal regulators in SAR and de-etiolation. Cross-species comparison of transcriptional regulation in SAR between *Arabidopsis* and maize identified shade-induced auxin biosynthesis as a possible target of domestication. Moreover, it was found that the distribution of shade- and FR-responsive genes was biased toward the maize1 subgenome, and this was associated with differential retention of light-responsive *cis*-elements between the two subgenomes, indicating that genome fractionation can occur both on genes and non-coding sequences with regulatory functions.

## Methods

### Plant material for RNA-seq experiments

To dissect the SAR in maize, the plant growth condition and treatment is the same as that used before to study *Arabidopsis* SAR [46]. Briefly, B73 maize plants were grown in a light-emitting diode growth chamber (Percival Scientific, Perry, IA) at 12 h light/12 h dark cycles at 24 °C under the following light outputs: Blue, 15 umol/m^2^s; Red, 96 umol/m^2^s; Far-red, 21 umol/m^2^s. On the fourth day after germination (at V1 stage), shoots were harvested above the upper node of the mesocotyl 3 h after lights were on. Then light outputs were immediately adjusted to: Blue, 15 umol/m^2^s; Red, 12 umol/m^2^s; Far-red, 105 umol/m^2^s. Samples were then harvested 1 h, 3 h, and 6 h after the onset of the simulated shade treatment. For each time point, six plants were pooled for each of the three biological replicates. To dissect the de-etiolation under FR, the plant growth condition and treatment is similar to that used in two previous studies [47, 61]. Briefly, B73 plants were grown in darkness at 24 °C. Four days after germination (etiolated V1 stage seedlings), shoots were harvested above the upper node of the mesocotyl. Remaining seedlings were immediately irradiated with 6 umol/m^2^s FR. Samples were then harvested after 1 h, 3 h, and 6 h treatment. For each time point, six plants were pooled for each of the three biological replicates.

### RNA-seq library construction, sequencing and analysis

RNA-seq libraries were generated from 2–5 μg total RNA and size-selected for a 250–300 bp insert for paired-end (PE) sequencing (100 bp for each end). Libraries were quantified on an Agilent bioanalyzer (Agilent, Santa Clara, CA) and sequenced using the Illumina HiSeq2000 system using standard Illumina protocols (Illumina Inc., San Diego, CA). The raw reads were trimmed and filtered using Fastx-toolkit (version 0.0.13). Tophat (version 2.0.12) was used to align reads to the maize reference genome (AGP v3.23). Gene-level expression values are represented by Reads Per Kilobase per Million (RPKM). Differentially expressed genes were identified using the exact test of Robinson and Smyth for two-group comparisons implemented in the EdgeR package [105]. A False Discovery Rate (FDR) corrected *p*-value < =0.05 and a threshold fold change > =2 were used to call differentially transcribed genes.

### Quantitative RT–PCR

Total RNA was isolated using Trizol (Invitrogen, Carlsbad, CA) according to the manufacturer’s instructions. Isolated RNA was treated with RNase-free DNase and purified using RNeasy mini columns (Qiagen, Hilden, Germany). First-strand cDNA was synthesized using SuperScript III reverse transcriptase (Invitrogen, Carlsbad, CA). The *tublin* transcript was used as an internal control to normalize the RNA quantity. Three biological replicates were included in quantitative RT–PCR analysis.

### Functional classification

Maize gene annotations using GO terms were acquired using the BLAST2GO pipeline, combined with existent GO annotation retrieved from Gramene BioMart. Maize genes were also assigned with MapMan bins based on their homology with rice and *Arabidopsis* genes using the Mercator pipeline [106].

### Cluster and functional enrichment analysis

Cluster analysis was applied to genes showing differential expression in at least one comparison in SAR or de-etiolation. Log2-transformed ratios were subjected to cluster analysis. For K-means clustering, the K-Means/K-Medians Support (KMS) module in the MEV program (http://www.tm4.org/mev.html) was used for clustering of DTGs. The number of clusters was determined by the Figures of Merit (FOM) module. Genes in each cluster were then classified into Mapman functional categories, and the Fisher’s exact test was used to test for enrichment of each functional category. FDR is controlled by Benjamini and Hochberg’s procedure. Differentially expressed transcription factors were clustered using the Self Organizing Tree Algorithm (SOTA) embedded in the MEV program.

### Cis-regulatory motif enrichment analysis

To determine enriched *cis*-regulatory elements on promoters of co-expressed genes, proximal promoter was defined as 2 kbp upstream and 500 bp downstream of transcription start site (TSS) since this region is adequate to capture the 5′ UTR and the first intron of most maize gene models. A comprehensive collection of plant position weight matrices (PWMs) [107] and also PWMs deposited in the JASPAR database were used. Promoters were scanned for significantly enriched *cis*-elements using the PWMEnrich package.

### Correlation network

The expression correlation network was calculated with Pearson correlation values between genes as edge weights. The resulting weighted network was clustered with the Markov cluster algorithm (MCL) using clusterMaker2 with granularity parameter 2 and default advanced settings [108]. The graph was visualized in Cytoscape version 3.2.1.

### Availability of data and materials

The datasets supporting the conclusions of this article are available in the SRA repository (accession SRP068070).

## Additional files

Additional file 1: Figure S1. Maize seedlings (inbred line B73) were grown under high R/FR and low R/FR (simulated shade) for 10 days. Note that seedlings grown under the simulated shade were with longer leaves and accumulated less anthocyanin. (PDF 131 kb) Additional file 2: Figure S2. Principle Component Analysis (PCA) of samples using genome-wide gene expression values. (PDF 192 kb) Additional file 3: Table S1. K-means clustering of DTGs. (XLSX 298 kb) Additional file 4: Table S2. Confirmation of RNA-Seq data by qRT-PCR. (XLSX 18 kb) Additional file 5: Table S3. Enriched *cis*-elements in each DTG cluster. (XLSX 29 kb) Additional file 6: Table S4. A summary of differentially transcribed transcription factors. (XLSX 28 kb) Additional file 7: Figure S3. Phylogenetic analysis of a group of early shade-induced *Arabidopsis* homeodomain-leucine zipper genes and their two putative maize orthologs. The evolutionary tree was inferred with amino acid sequences using the Neighbor-Joining method implemented in MEGA6. Bootstrap values (with 100 replicates) were shown next to the branches. The tree was rooted on a close homolog in *P. patens.* (PDF 100 kb) Additional file 8: Figure S4. Phylogenetic analysis showed a single maize ortholog of the early shade-inducible *Arabidopsis* atypical basic helix-loop-helix (bHLH) genes *PAR1* and *PAR2*. The evolutionary tree was inferred with amino acid sequences using the Neighbor-Joining method implemented in MEGA6. Bootstrap values (with 100 replicates) were shown next to the branches. The tree was rooted on a close homolog in *P. patens.* (PDF 97 kb) Additional file 9: Figure S5. Phylogeny and structure of putative maize PIFs. Seven maize bHLH family transcription factors were identified as PIFs, due to their close relationship with *Arabidopsis* PIFs in the phylogenetic tree (a), and also the conserved ABP motif shared among all PIFs responsible for their physical interaction with phytochrome B (b). Phylogenetic analysis was conducted by MEGA6 using the neighbor-joining method. Bootstrap values were obtained using 100 bootstrap replicates and are shown next to the branches. The tree was rooted on the closest homolog of *Arabidopsis* PIFs in *P. patens*. Note that HFR1 and PIL1 are on a branch (shaded in blue) without any maize orthologs. (PDF 129 kb) Additional file 10: Table S5. Conserved inter-species expression patterns in SAR. (XLSX 11 kb) Additional file 11: Table S6. Contrasting inter-species expression patterns in SAR. (XLSX 10 kb) Additional file 12: Table S7. Enriched GO terms for shade-responsive DTGs. (XLSX 13 kb) Additional file 13: Table S8. A comparison of light-responsive genes between the two subgenomes of maize. (XLSX 24 kb)

## Abbreviations

CHI: chalcone flavanone isomerase
CHS: chalcone synthase
*COP1*: *constitutive photomorphogenic1*
DTG: differentially transcribed gene
DTTF: differentially transcribed TF
EE: evening element
*ETC3*: *enhancer of try and CPC3*
F3H: flavanone 3-hydroxylase
FDR: false discovery rate
*FKF1*: *flavin-binding, kelch repeat, F-BOX1*
FLS: flavonol synthase
FR: far-red
*GL3*: *GLABROUS 3*
*HFR1*: *long hypocotyl in far-red1*
*HY5*: *elongated hypocotyl5*
*HYH*: *HY5-HOMOLOG*
*LAF1*: *long after far-red light1*
MCL: Markov cluster algorithm
*OZF*: *oxidation-related zinc finger*
*PAP1*: *phytochrome-associated protein1*
PAR: photosynthetically active radiation
PE: paired-end
*PIF*: *phytochrome-interacting factor*
PWMs: position weight matrices
R: red
RPKM: reads per kilobase per million
*RSM2*: *radialis-like SANT/MYB 2*
SAR: shade avoidance response
SOTA: self organization tree algorithm
*TAA1*: *tryptophan aminotransferase of arabidopsis1*
*tb1*: *teosinte branched1*
*tga1*: *teosinte glume architecture1*
TSS: transcription start site
UPR: unfolded protein response
