# Supplementary figures and images for: Regulatory modules controlling early shade avoidance response in maize seedlings
Source: BMC Genomics. 2016 Mar 31;17:269. doi: 10.1186/s12864-016-2593-6 (PMC4815114; doi:10.1186/s12864-016-2593-6)

**Fig. S1**

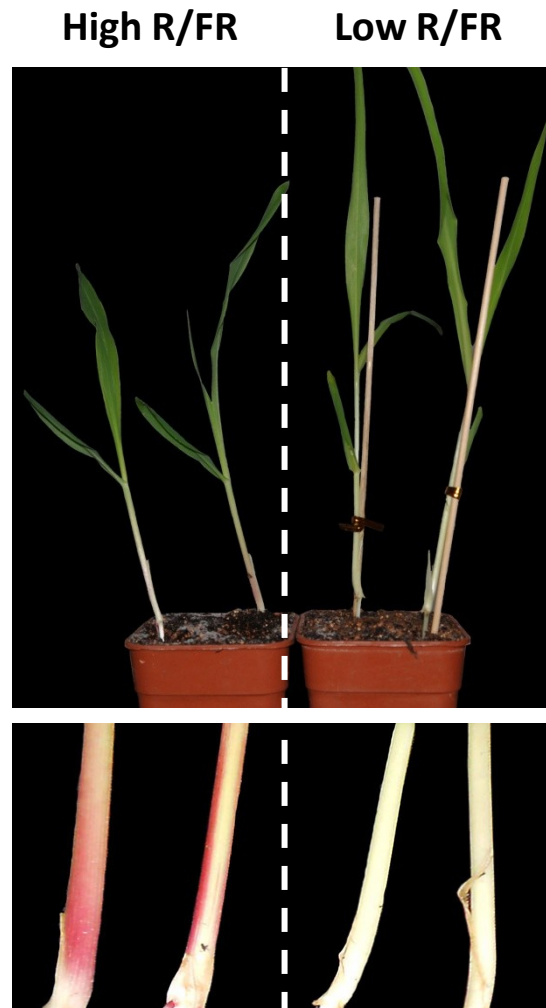

Supplement: Additional file 1: Figure S1. — Maize seedlings (inbred line B73) were grown under high R/FR and low R/FR (simulated shade) for 10 days. Note that seedlings grown under the simulated shade were with longer leaves and accumulated less anthocyanin. (PDF 131 kb) [file 12864_2016_2593_MOESM1_ESM.pdf]

Fig. S2

**a**      *shade avoidance response*

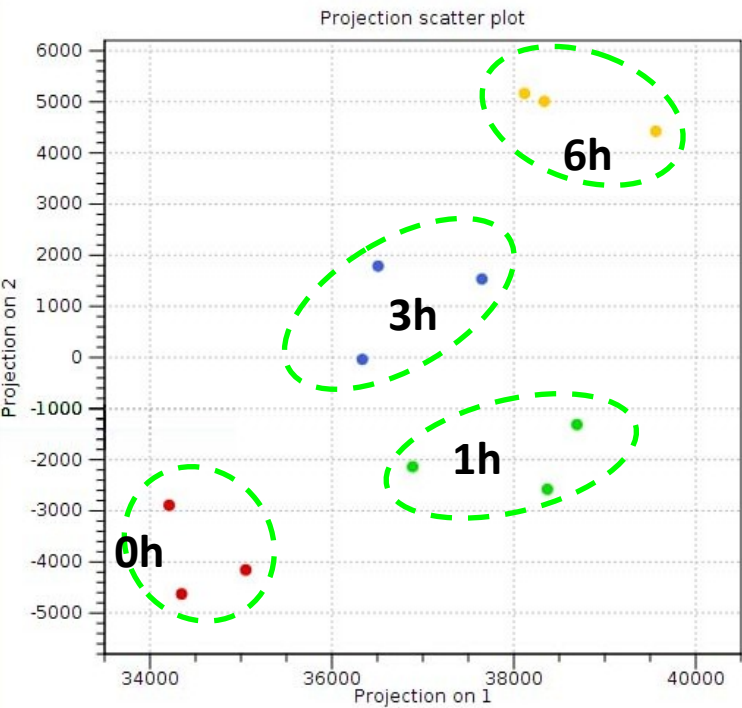

**b**      *deetiolation under FR*

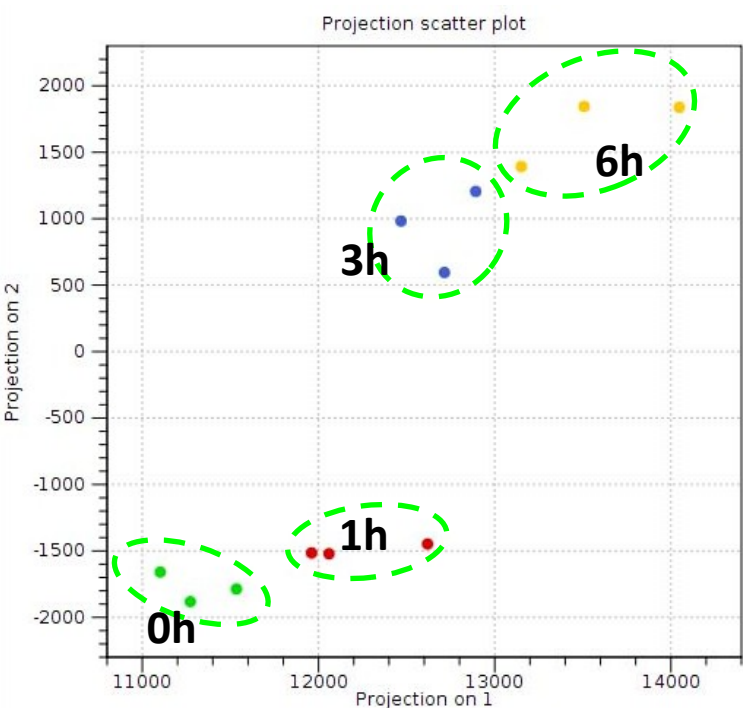

Supplement: Additional file 2: Figure S2. — Principle Component Analysis (PCA) of samples using genome-wide gene expression values. (PDF 192 kb) [file 12864_2016_2593_MOESM2_ESM.pdf]

**Fig. S3**

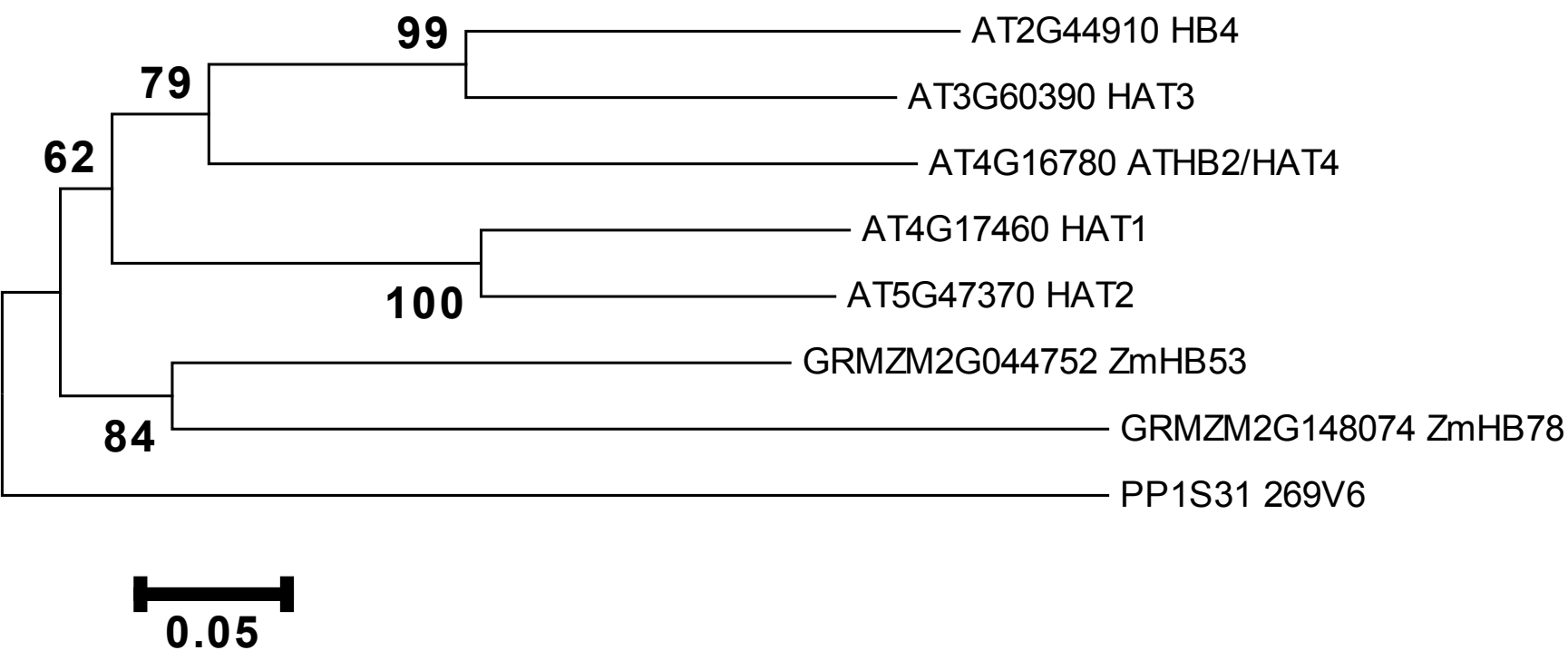

Supplement: Additional file 7: Figure S3. — Phylogenetic analysis of a group of early shade-induced Arabidopsis homeodomain-leucine zipper genes and their two putative maize orthologs. The evolutionary tree was inferred with amino acid sequences using the Neighbor-Joining method implemented in MEGA6. Bootstrap values (with 100 replicates) were shown next to the branches. The tree was rooted on a close homolog in P. patens. (PDF 100 kb) [file 12864_2016_2593_MOESM7_ESM.pdf]

Fig. S4

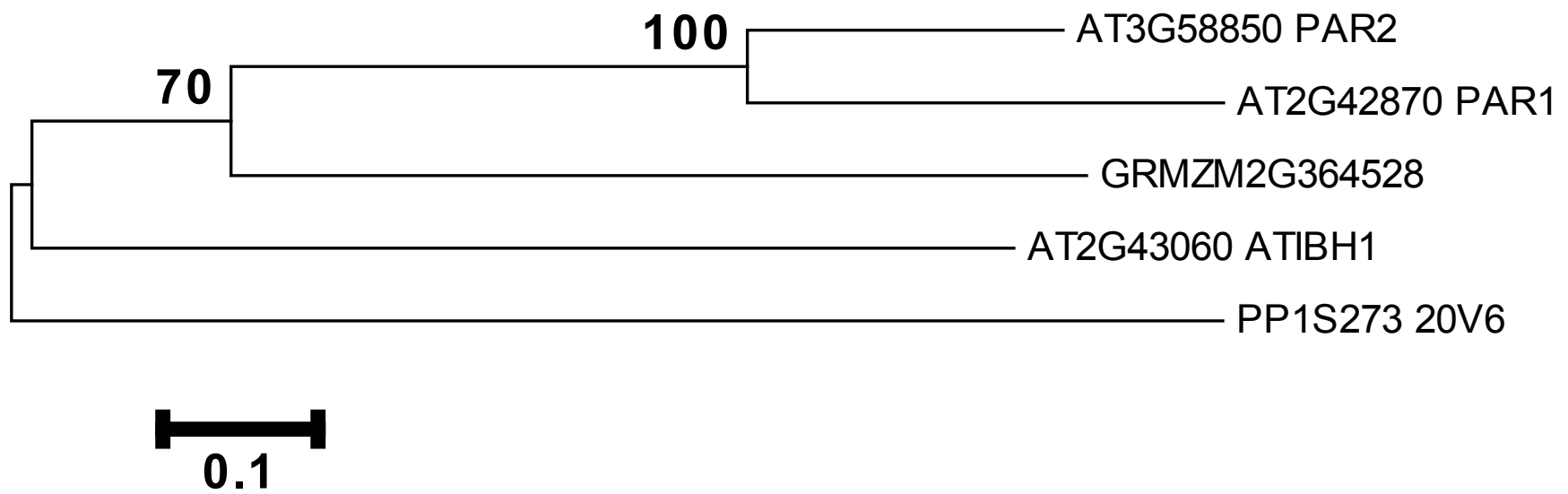

Supplement: Additional file 8: Figure S4. — Phylogenetic analysis showed a single maize ortholog of the early shade-inducible Arabidopsis atypical basic helix-loop-helix (bHLH) genes PAR1 and PAR2. The evolutionary tree was inferred with amino acid sequences using the Neighbor-Joining method implemented in MEGA6. Bootstrap values (with 100 replicates) were shown next to the branches. The tree was rooted on a close homolog in P. patens. (PDF 97 kb) [file 12864_2016_2593_MOESM8_ESM.pdf]

Fig. S5

a

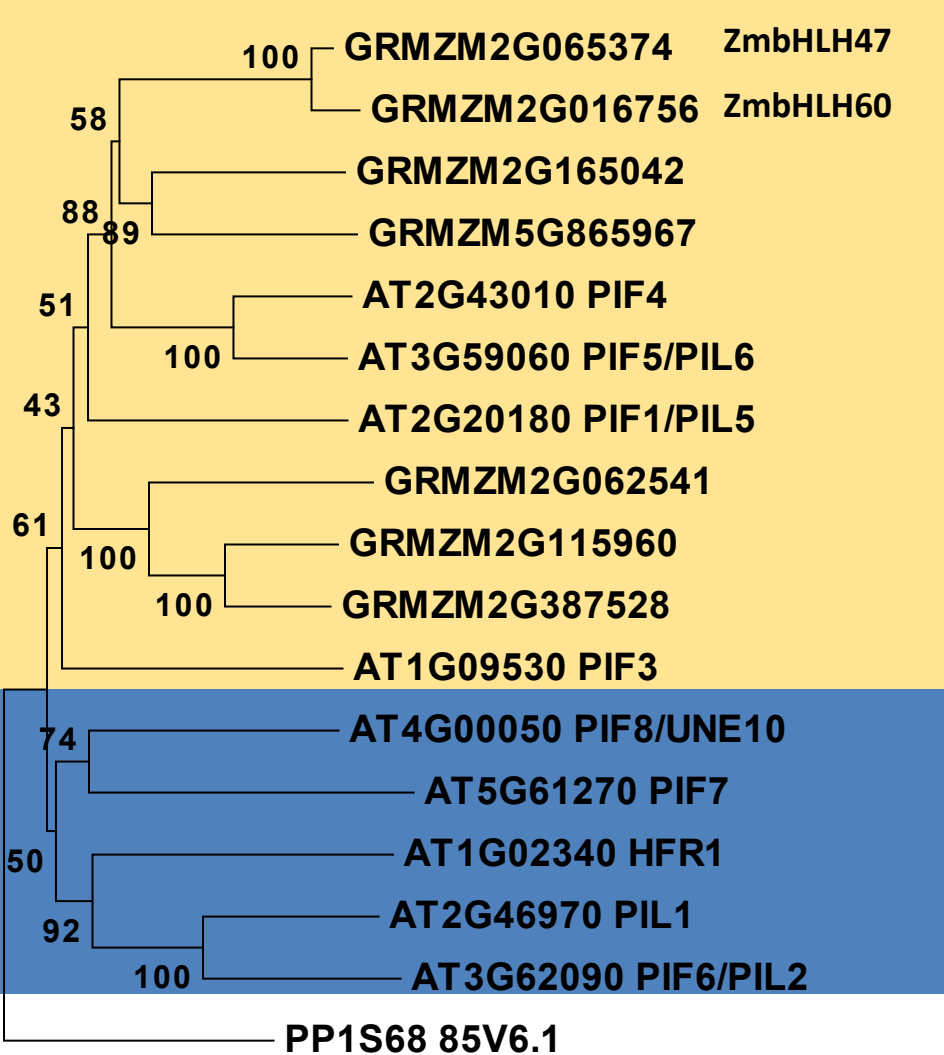

0.1

b

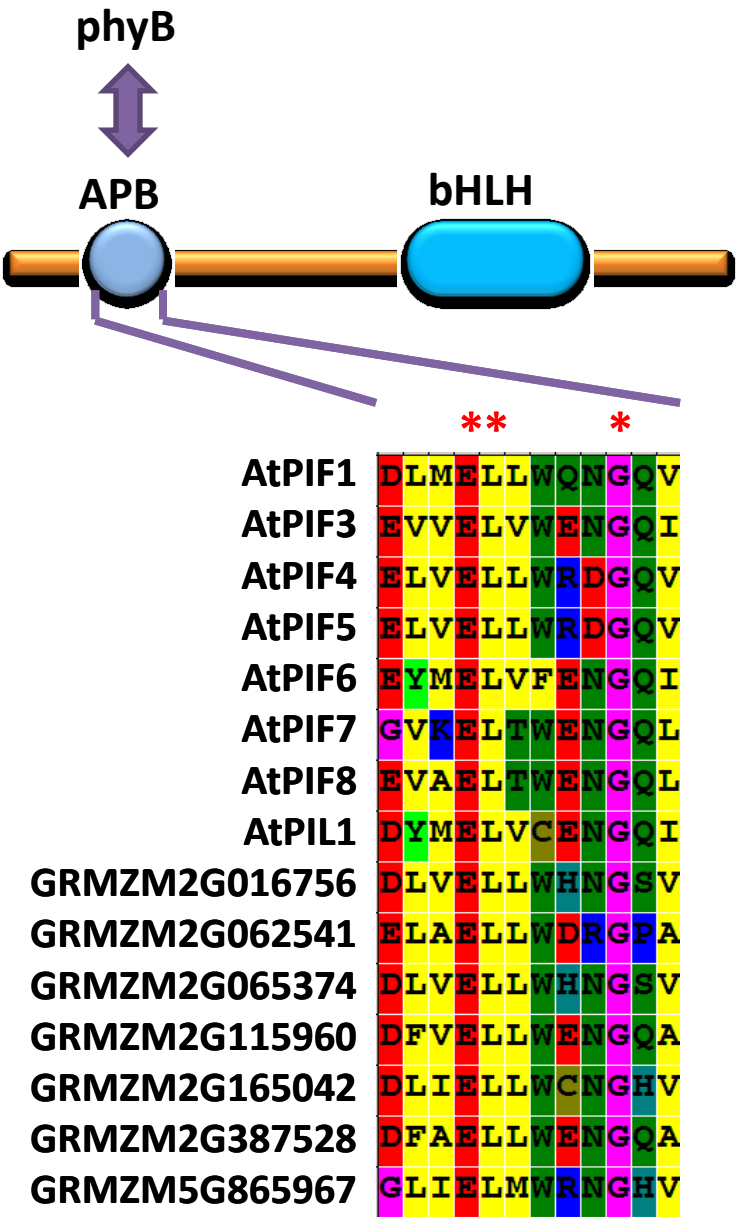

Supplement: Additional file 9: Figure S5. — Phylogeny and structure of putative maize PIFs. Seven maize bHLH family transcription factors were identified as PIFs, due to their close relationship with Arabidopsis PIFs in the phylogenetic tree (a), and also the conserved ABP motif shared among all PIFs responsible for their physical interaction with phytochrome B (b). Phylogenetic analysis was conducted by MEGA6 using the neighbor-joining method. Bootstrap values were obtained using 100 bootstrap replicates and are shown next to the branches. The tree was rooted on the closest homolog of Arabidopsis PIFs in P. patens. Note that HFR1 and PIL1 are on a branch (shaded in blue) without any maize orthologs. (PDF 129 kb) [file 12864_2016_2593_MOESM9_ESM.pdf]
